# Influence of Clinical Factors on miR-3613-3p Expression in Colorectal Cancer

**DOI:** 10.3390/ijms241814023

**Published:** 2023-09-13

**Authors:** Paulina Gil-Kulik, Alicja Petniak, Natalia Kluz, Grzegorz Wallner, Tomasz Skoczylas, Aleksander Ciechański, Janusz Kocki

**Affiliations:** 1 Department of Clinical Genetics, Medical University of Lublin, 11 Radziwillowska Str., 20-080 Lublin, Poland; nataliakluz99@gmail.com (N.K.); janusz.kocki@umlub.pl (J.K.); 2 II Chair and Department of General and Gastrointestinal Surgery and Surgical Oncology of the Alimentary Tract, 16 Staszica Str., 20-081 Lublin, Poland; grzegorz.wallner@umlub.pl (G.W.); tomasz.skoczylas@umlub.pl (T.S.); aleksander.ciechanski@umlub.pl (A.C.)

**Keywords:** colorectal cancer, microRNA, gene expression regulation, miR-3613-3p

## Abstract

Colorectal cancer (CRC) is the second most common cause of cancer-related death globally. Because of a tendency to be an asymptomatic primary tumor and therefore resulting in late detection, most CRC patients are diagnosed in the advanced stage. Several miRNAs have the potential to become novel noninvasive biomarkers measured as diagnostic and prognostic indicators of CRC to guide surgical therapies and promote the understanding of the carcinogenesis of CRC. Since the change of miR-3613-3p was associated with several types of cancer other than colorectal cancer, there is a lack of functional evidence and the results are inconsistent. We conducted a pilot microarray study in which we noted a decreased expression of miR-3613-3p in colorectal cancer cells, then we confirmed the expression of miR-3613-3p by qPCR on a group of 83 patients, including 65 patients with colorectal cancer, 5 with a benign tumor and 13 from the control group. We noted that in both malignant and benign tumors, miR-3613-3p is downgraded relative to the surrounding tissue. As a result of the study, we also observed colorectal tumor tissue and surrounding tissue in patients with colorectal cancer who received radiotherapy before surgery, which showed a significantly higher expression of miR-3613-3p compared to patients who did not receive radiotherapy. In addition, we noted that the tissue surrounding the tumor in patients with distant metastases showed a significantly higher expression of miR-3613-3p compared to patients without distant metastases. The increased expression of miR-3613-3p in patients after radiotherapy suggests the possibility of using this miR as a therapeutic target for CRC, but this requires confirmation in further studies.

## 1. Introduction

Colorectal cancer (CRC) is the second most common cause of cancer-related death globally [1]. In 2020, there were an estimated 1.93 million new cases of CRC and an estimated 0.94 million disease-related deaths worldwide [2]. The advancements of treatments including surgery, chemotherapy, radiotherapy and targeted therapy improved the five-year survival rate of patients with CRC [3]. However, because of a tendency to be an asymptomatic primary tumor and therefore resulting in late detection, most CRC patients are diagnosed in the advanced stage [4].

CRC is staged using the American Joint Committee on Cancer (AJCC) tumor/node/metastasis (TNM) staging system. On the basis of the characteristics of the primary tumor (T), and the extent of regional lymph node involvement (N) and distant metastasis (M), stages are assigned. In addition, on the basis of preoperative clinical assessment (c) or pathologic evaluation of metastatic tissue (p), metastasis may be defined clinically or pathologically [5].

Prognostic and predictive routinely used factors include the clinical stage of CRC with the uncertain role of other potential factors. Colorectal carcinoma is traditionally classified according to cancer location (proximal colon, distal colon and rectum) histological type (mucinous versus non-mucinous; well-moderate versus poorly differentiated) and genetic features [6,7]. Molecular classification has become an integral component of pathologic evaluation. Currently, to better predict the behavior of a tumor, genetic alterations in *KRAS*, *v-RAF*, *BRAF* and *CDX2* are used to guide prognostication and optimize adjuvant treatment [8].

Given that morphology and molecular features are reliable to unify the correct CRC categorization, four consensus molecular subtypes (CMSs) were proposed by the Colorectal Cancer Subtyping Consortium: CMS 1 (representing 14% of CRC) as a kind of microsatellite instability (MSI)-high CRC (II), CMS 2 (37% of CRC) with extensive activation of WNT and MYC signaling pathways, CMS 3 (13% of CRC), which exhibits mostly KRAS-activating mutations, and CMS 4 (23% of all CRCs) as a type of tumor with remarkable stromal invasion related to epithelial mesenchymal transition (EMT). On that account, an accurate CRC-subtype classification system is crucial for basic research and clinical outcome [9,10,11,12].

Recently the knowledge of miRNA biology has distinctly increased. The role of miRNAs in different diseases, especially in cancer, has made miRNAs inviting targets for innovative therapies [13]. MicroRNAs with an average 22 nucleotides in length are released by healthy and cancerous cells [14,15]. MicroRNA constitute a negative-regulator class of small, non-coding, single-stranded RNAs that function as post-transcriptional regulates of gene expression, and are involved in many biological events [13,16]. The meaning of the specific expression signatures as potential blood-, stool- or urine-based diagnostic markers was important, especially in cancers where other early discovery methods do not exist. [14]. Furthermore, the advantages of miRNAs as biomarkers include: non-invasive, reproducible methods, and huge stability miRNAs in serum and plasma [17].

For colorectal cancer, plenty of relevant microRNAs and their mechanisms were disclosed [18]; for example, highly expressed miRNA-200 induced epithelial mesenchymal transition (EMT), thus increasing the invasiveness and metastatic activity of CRC [19], a high expression of miR-762 attenuated cell growth and invasion, which might be accompanied by increased Wnt/beta catenin signaling [20], higher levels of transcribed miR-92a played a pivotal role in CRC by interacting with anti-apoptotic molecule *BCL-2* and contributing to the survival of cancerous cells [21]; and metastasis of colorectal cancer is also promoted by miR-6716-5p through downregulating *NAT10* expression [22]. In case of microRNA-96, pro-proliferative and anti-apoptotic mechanism in CRC was revealed by downregulating *AMPKα2* and upregulating *FTO* and *MYC* in the *AMPKα2/FTO/m6A/MYC* pathway [23].

Thus, miRNAs have the potential to become novel, noninvasive biomarkers measured as diagnostic and prognostic indicators of CRC to guide surgical therapies and promote the understanding of the carcinogenesis of CRC.

A change of miR-3613 was associated with several types of cancer other than colorectal cancer, however, there is lack of functional evidence and the results are inconsistent [24,25,26].

In our study, we focused on evaluating miR-3613-3p expression in colorectal cancer cells. Initially, we selected this miRNA as significant by the array technique, and then the aim of our study was to confirm it by using the qPCR technique and determine the effect of the colorectal cancer stage on the expression level of miR-3613-3p.

## 2. Results

Based on the study, it was observed that the colorectal tumor, the tissue surrounding the tumor and normal intestinal tissue, express miR-3613-3p.

**Figure 1:**
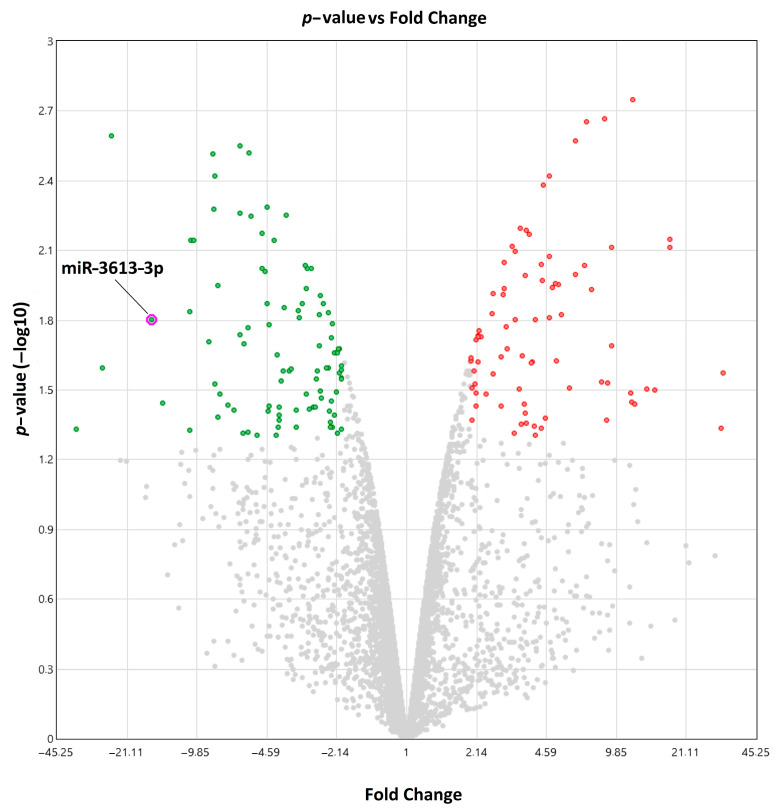
Volcanic diagram of colorectal tumor microRNA expressions between colon adenocarcinoma patients and surrounding tissue. Red dots—microRNAs expressed significantly more than twice in the tumor tissue compared to the tissue surrounding the tumor; green dots—microRNAs expressed significantly less than twice in the tumor tissue compared to the tissue surrounding the tumor; gray dots—microRNA expressed in tumor tissue compared to tissue surrounding the tumor (*p* > 0.05).

The pilot microarray study showed a mean expression in the tumor (log2) of 0.85, while the mean expression in the surrounding tissue (log2) was 4.86, at *p* = 0.0158, thus a fold change of −16.14 was found (Figure 1).

**Figure 2:**
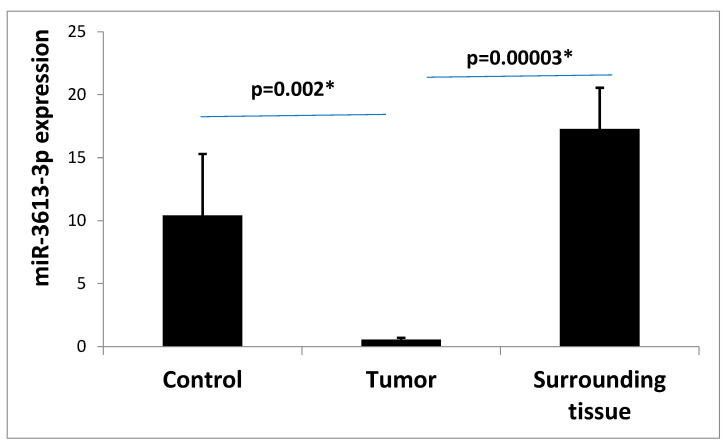
Mean expression (RQ ± SE) of miR-3613-3p in the surrounding tissue depending on depending on the analyzed group: control, tumor, surrounding tissue (only patients without radiotherapy). * *p* < 0.05 Kruskal–Wallis ANOVA.

We confirmed the results with qPCR and observed that colorectal cancer cells tended to have lower miR-3613-3p expression values compared to the surrounding tissue (*p* = 0.00003) and control tissue (*p* = 0.002) (Figure 2).

**Figure 3:**
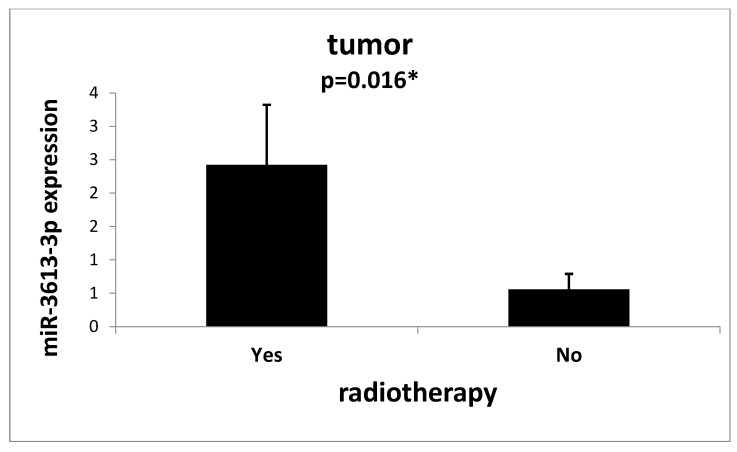
Mean expression (RQ ± SE) of miR-3613-3p in the tumor depending on whether the patient had radiotherapy before surgery to remove the tumor. * *p* < 0.05 U Mann–Whitney Test.

**Figure 4:**
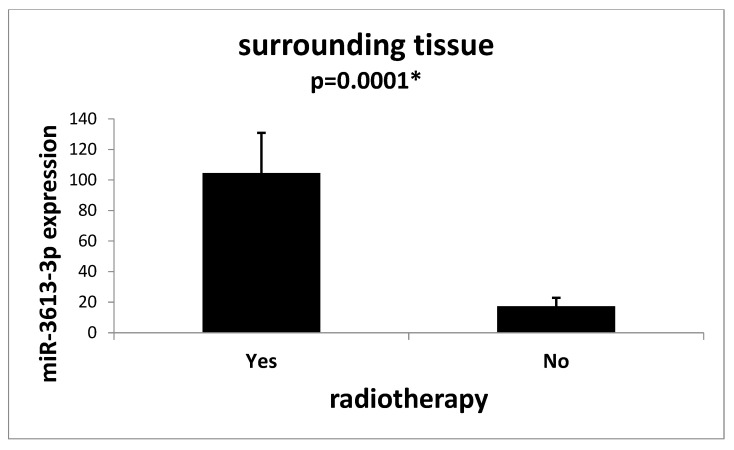
Mean expression (RQ ± SE) of miR-3613-3p in the surrounding tissue depending on whether the patient had radiotherapy before surgery to remove the tumor. * *p* < 0.05 U Mann–Whitney Test.

Moreover, we observed that both the tumor tissue (*p* = 0.016) (Figure 3) and the tissue surrounding the tumor (*p* = 0.0001) (Figure 4) of patients who underwent radiotherapy prior to removal surgery had an increased expression of miR-3613-3p.

**Figure 5:**
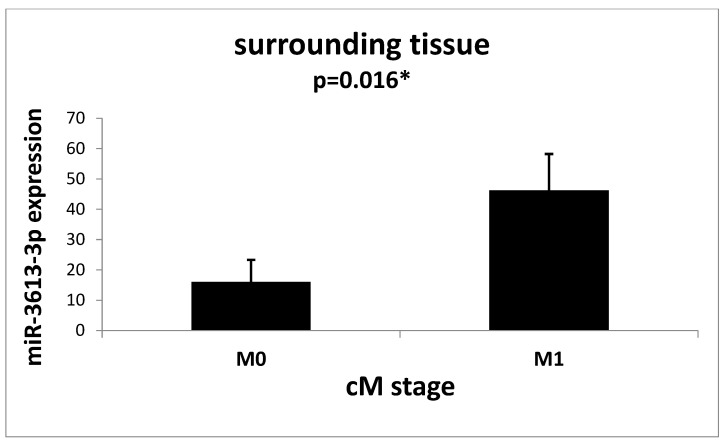
Mean expression (RQ ± SE) of miR-3613-3p in the surrounding tissue depending on the cM stage. M0 vs. M1 (only patients without radiotherapy) * *p* < 0.05 U Mann–Whitney Test.

Analyzes of the relationship between the expression of miR-3613-3p and the stage of the TNM tumor showed no significant relationships; we only noticed some trends that were denied in the Supplementary Materials. It was shown that the expression of the examined microRNA in the cells of the tissue surrounding the tumor in patients with metastases to distant organs (cM1 stage) is statistically and significantly higher compared to the cells of the tissue surrounding the tumor obtained from patients from the M0 group (*p* = 0.016) (Figure 5).

**Figure 6:**
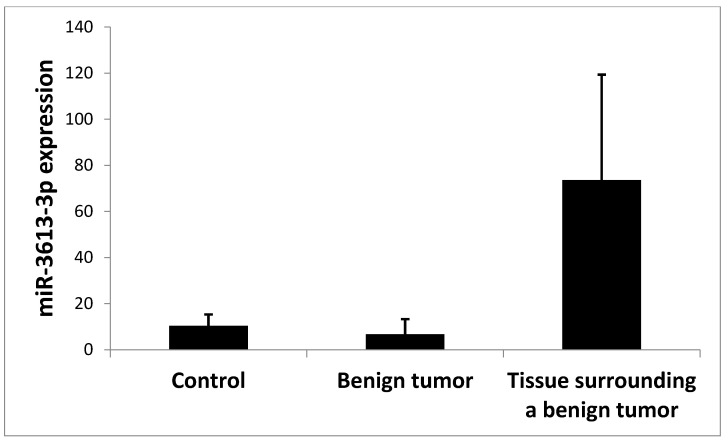
Mean expression (RQ ± SE) of miR-3613-3p depending on the analyzed group: control, benign tumor, tissue surrounding a benign tumor. *p* < 0.05 Kruskal–Wallis ANOVA.

In addition, when analyzing several samples of benign tumors, we also observed that the expression of miR-3613-3p in these tumors was statistically and significantly lower compared to the tissue surrounding the benign tumor (*p* = 0.026); no significant difference was observed in the expression of miR-3613-3p in the benign tumor compared to the control, and there was also no significant difference between the tissue surrounding the benign tumor and the control tissue (Figure 6). MiR-3613-3p expression in a benign tumor was shown to be statistically and significantly higher than in a malignant tumor (*p* = 0.02) (Table 1).

## 3. Discussion

Colorectal cancer was the third most commonly diagnosed cancer type in the world. It was the third most common cancer in men, but the second most common cancer in women, after breast cancer [27]. Since many patients at the moment of diagnosis of CRC are in the advanced stage of the disease, often with the presence of metastases or in the incurable stage of cancer, it seems necessary to identify new molecular prognostic factors in order to predict the course of the disease or response to therapeutic intervention in these patients.

An increasing number of studies are focused on evaluating the impact of miRNAs on tumorigenesis. A huge number of miRNAs are regarded as oncogenes or tumor suppressors. miRNAs play a systematic role in CRC proliferation, metastasis, angiogenesis, autophagy, apoptosis, and chemoradiotherapy, regulating multiple-related signaling pathways [28].

In our work, we assessed the role of miR-3613-3p in colorectal adenocarcinoma. As far as we are aware, we are the only ones to show the level of miR-3613-3p not only in tumor cells and in the tissue surrounding the tumor, but also in normal control tissue. In addition, we also examined a benign tumor of the intestine and its surrounding tissue. Our studies showed that the expression of miR-3613-3p in malignant and benign tumor cells was significantly lower compared to the tissue surrounding these tumors. We also noted that in patients who received radiotherapy before surgery to remove the tumor, the expression of miR-3613-3p in the tumor tissue and in the tissue surrounding the tumor was significantly higher compared to patients who did not receive radiotherapy. In addition, in our study, we noted that the expression of miR-3613-3p in the tissue surrounding colorectal cancer in patients with distant metastases at the cM1 stage was significantly higher compared to patients at the M0 stage.

There are few studies evaluating the role of miR-3613-3p in CRC. Several studies have reported the change of miR-3613-3p associated with several types of cancer, including colon cancer [29], gastric cancer [30], breast cancer [31], lung cancer [32] and ovarian cancer [33], but the results are inconsistent and lack functional evidence. MiR-3613-3p was first identified in extracellular vesicles from the human colon carcinoma cell line [34].

In a breast cancer study, Chen et al. proved the important suppressive role of miR-3613-3p in breast cancer progression. They demonstrated the important role of miR-3613-3p as an innovative prognostic marker or therapeutic target in breast cancer patients. They observed that miR-3613 *locus* was frequently deleted in the breast cancer tissue and its deletion was associated with the breast cancer subtypes and poor prognosis in estrogen-receptor-positive patients [31].

In the case of hepatocellular carcinoma, Zhang et al. showed that miR-3613-3p affects cell proliferation and the cell cycle of tumor cells. For hepatocellular carcinoma, Zhang et al. showed that miR-3613-3p affects cell proliferation and the cell cycle of tumor cells. An overexpression of miR-3613-3p in the human liver cancer cell, HepG2, transfected with a hsa-miR-3613-3p mimic showed a reduction in cell viability at 1-4 days after transfection, compared with that at day 0 [35].

The study examining changes in miR-3613-3p expression in colorectal cancer was conducted by Yan et al. on two colon cancer cell lines. SW480, derived from primary colon carcinoma, and SW620, derived from lymph node metastasis, which were obtained from the same patient. Mir-3613-3p in SW620 cells in contrast to SW480 cells was downregulated [29]. Xiag et al. found that miR-3613-3p was under-expressed in CRC [36]. Also, Avsar et al., in their study, showed that miR-3613-3p was downregulated in tissues with CRC [37]. The results of the study presented by Yan, Xiang and Avsar correspond with the results obtained in our study, where we also showed a reduction of miR-3613-3p expression in tumor cells compared to the tissue surrounding the tumor. In addition, Avsar did not observe any relationship between miR-3613-3p expression and clinical pathological features of the tumor in its studies, which is also consistent with our observations.

The results of miR-3613-3p expression studies in various cancers presented in the literature are not consistent, however, there are studies showing both an increase in expression and a decrease in various types of cancer, which definitely makes it difficult to correctly determine the function of this microRNA in the formation of cancer.

Bibi et al. investigated the role of miRNAs in gastric cancer patients and presented a profile of gastric cancer miRNAs in which many miRNAs, including miR-3613-3p, were aberrantly expressed from normal tissues, suggesting their involvement in the development and progression of gastric cancer. Their results showed that a significant up-regulation of miR-3613-3p was markedly observed in early stage and late-stage GC compared to normal control [30].

Also, a study by Liu et al. showed that activated fibroblast exosomes with high levels of miR-3613-3p play an oncogenic role in breast cancer cell survival and metastasis. suggesting that miR-3613-3p functions as a therapeutic target [38].

Increased expression of miR-3613-3p in cancer cells was also demonstrated by Fricke et al. In their studies. miR-3613-3p was shown to be significantly upregulated (fold change: >2.5; *p* < 0.05) in patients with dedifferentiated liposarcoma, as compared to healthy controls [39].

In studies on miRNA regulation in ovarian cancer and endometriosis, Wu et al. showed the expression of miR-3613 as having a significant up-regulation in ovarian tumors compared to their benign endometriosis. In addition, miR-3613-3p down-regulate *PTEN*, and the regulator of *PI3K-Akt* signaling a downstream of *EGFR* [40].

Song et al., in their lung adenocarcinoma study, reported that miR-3613-3p regulates EGFR signaling genes in the epithelial-mesenchymal transition of lung adenocarcinoma [41].

*EGFR* overexpression is found in 25 to 82% of colorectal cancer cells and is a useful target in the treatment of colorectal cancer metastasis [42]. In the pathogenesis of CRC, the *EGFR* signaling pathway is implicated in the promotion of tumor cell proliferation, migration, inhibition of apoptosis, angiogenesis, and immune evasion by tumor cells [43]. EGFR-targeted therapy was confirmed to increase overall survival by 10–20% in colorectal cancer. With the emergence of *KRAS* and *BRAF* mutations, resistance to EGFR-targeted therapy has developed, driven by mechanisms affecting both cellular pathways and the tumor microenvironment [44]. The kinase-activating mutations that result in enhanced EGFR tyrosine kinase activity might occur as a result of, or in addition to, anti-EGFR therapy [42]. Taking into account the influence of miR-3613-3p on EGFR demonstrated by other researchers, and the fact that CRC is very often characterized by an overexpression of *EGFR*, it can be speculated that the relationship of miR-3613-3p expression provides a good introduction to further studies evaluating the therapeutic potential of the tested microRNA.

The role of miR-3613-3p in the pathomechanism of cancer remains unclear; the literature has shown both an increase in expression and a decrease in expression or deletion of miR-3613-3p in cells of various types of cancer. In most of the cited studies, miR-3613-3p is perceived as a suppressive microRNA, and we also agree with this theory. Chen et al. suggested that miR-3613-3p can inhibit sphere proliferation and formation, and promote apoptosis in vitro and inhibit tumor growth and metastasis in vivo, thus ultimately suggesting that it can regulate the cell cycle by targeting *SMS*, *PAFAH1B2* or *PDK3* to stop tumor progression [31].

In our study, we noticed that the tissue surrounding the tumor shows a higher expression compared to the normal control tissue of the intestine. An increased expression of miR-3613-3p in the zone around the tumor may have a potential effect in inhibiting its growth and development. Moreover, the significantly increased expression of miR-3613-3p in the tissue surrounding the tumor, taken from patients with distant metastases, as compared to patients without metastases, seems to confirm a certain protective function of the surrounding tissue.

Many authors see miR-3613-3p as a potential therapeutic target. Among others, research by Yu et al. showed that an overexpression of miR-3613-3p in cancer tissues of patients was clinically and pathologically correlated with favorable prognosis; researchers also showed that in vitro overexpression of miR-3613-3p inhibited cell proliferation, induced G1 cell cycle arrest and increased cell sensitivity for treatment [45].

In our studies, we noticed that miR-3613-3p is overexpressed in patients after radiotherapy, compared to patients who did not receive radiotherapy. The demonstrated relationship shows that miR-3613-3p can be perceived as a potential therapeutic target, but it should be remembered that our results, although they are statistically significant, were carried out only on a group of five patients with radiation therapy, so they require confirmation on a larger group.

In addition, in our research, we examined several samples from patients with benign colorectal lesions. In this analysis, we noted that the benign tumor shows a significantly higher expression of miR3613-3p than the malignant tumor, but lower expression than the surrounding tissue. The expression in the benign tumor is not significantly different from the expression in the control tissue. Interestingly, both in the case of a malignant and benign tumor, the expression in their surrounding tissue is significantly higher compared to the tumor, while there are no significant differences in the ratio of the surrounding tissue to the control. It seems that the tissue surrounding the tumor has a specific function in the context of the expression of miR-3613-3p as a suppressive microRNA, especially since the surrounding tissue is a tumor control in many studies, but this is only a suggestion that needs to be confirmed in further research.

## 4. Materials and Methods

### 4.1. Sample Collecting

The study involved 153 samples of tumor tissue, surrounding tissue and healthy mucosa tissue collected during surgeries due to colorectal cancer. In total, 65 tumors were pathologically diagnosed as primary colorectal adenocarcinoma, and 5 were diagnosed as benign tumors. The patients were hospitalized at the Second Department of General. Gastroenterological and Gastrointestinal Surgery SPSK1 in Lublin. Poland and then transferred to the Department of Clinical Genetics Medical University of Lublin. The patients participating in the study provided their informed consent and the study was approved by the Ethics Committee of the Medical University of Lublin (decision number: KE-0254/12/2018). The research was carried out in accordance with the Helsinki Declaration.

For the experiments, 65 fragments of colorectal tumor and surrounding tissue were used taken from patients aged 42 to 80; the mean age in the study group was 65.8 ± 9.27 years, including 44 men and 21 women. In the group of 65 patients with a malignant tumor, 5 patients had chemotherapy prior to surgery to remove the colorectal tumor.

In the group of 5 patients with a benign tumor, the mean age of patients was 60 ± 8.9 years.

In the group of patients with a malignant tumor without prior radiotherapy, the average tumor size was 4.52 ± 1.97 cm, whereas in the group of patients with a benign tumor, the average tumor size was 3.8 ± 1.3 cm, while the average tumor size in patients who underwent radiotherapy before surgery was 2.67 ± 0.6 cm.

The control group was normal colon tissue taken from 13 patients with benign lesions the fragment healthy mucosa tissue was taken at the appropriate distance from the benign lesion. The age of the patients in the control group ranged from 55 to 76 years; the mean age was 66.72 ± 7.9 years including 7 men and 6 women.

The characteristics of the study sample are presented in Table 2, Table 3, Table 4 and Table 5.

### 4.2. Total RNA Isolation

The collected tissue fragments were homogenized using a Precellys 24 homogenizer (Bertin-Instruments, Montigny-le-Bretonneux, France). Tissue disintegration was achieved by using stainless steel balls (TK Biotech, Warsaw, Poland) placed in the homogenized biological material.

Total RNA was successfully extracted from 140 tumor samples and 13 healthy mucosa tissues using the RNeasy Micro Kit (Qiagen, Valencia, CA, USA), according to manufacturer’s protocol. The quantity and quality assessment of isolated total RNA was performed using NanoDrop 2000c Spectrophotometer (Thermo Fisher Scientific, Waltham, MA, USA) and Bioanalyzer 2100 (Agilent Technologies, Santa Clara, CA, USA) and an Agilent RNA 6000 Nano Kit, according to the manufacturer’s protocol.

### 4.3. Reverse Transcription

Approximately 10 ng total RNA was used for cDNA synthesis. The reverse transcription process was performed according to the manufacturer’s protocol using the TaqMan™ MicroRNA Reverse Transcription Kit (Applied Biosystem, Foster City, CA, USA) and miRNA-specific stem-loop primers (Applied Biosystem, Vilnius, Lithuania). cDNA was synthesized in reverse transcription PCR (RT-PCR) using the Veriti 96-Well Thermal Cycler (Applied Biosystems, Foster City, CA, USA). The reactions were incubated for 30 min in 16 °C, 30 min in 42 °C and 5 min in 85 °C (stop reaction).

### 4.4. qPCR Reaction

Quantitative real-time PCR was run on the StepOnePlus Real-Time PCR System (Applied Biosystems, Waltham, MA, USA) using and standard TaqMan^®^ MicroRNA Assays (Applied Biosystems by Thermofisher Scientific, Pleasanton, CA, USA): hsa-miR-3613-3p (Assay ID 463183_mat, mature miRNA sequence: ACAAAAAAAAGCCCAACCCUUC) and RNU48 (Assay ID: 001006, control sequence: GATGACCCCAGGTAACTCTGAT-GTGTCGCTGATGCCATCACCCAGCGCTCTGAC) as a control. Each sample amplification was performed in triplicate in a final volume of 10 μL, including 0.67 μL of RT product along with 3.83 ul nuclease–free water and 0.5 μL miRNA-specific primer/probe mix and 5 μL TaqMan™ Universal Master Mix II, with UNG (Applied Biosystems, Vilnius, Lithuania). The reactions were incubated in a 96-well MicroAmp Fast Optical 0.1 mL reaction plate at 50 °C for 2 min (Uracil-N glycosylase incubation), 95 °C for 10 min (polymerase activation), and 40 cycles of 95 °C for 15 s (denature) and 60 °C for 1 min (anneal/extend).

The expression of the examined miRNAs was calculated from the formula RQ = 2^−ΔΔCT^ [46].

RNU48 snRNA was used as a normalizer during the analysis. The ExpressionSuiteSoftware Version 1.3 (Life Technologies, Waltham, MA, USA) was used to calculate miRNA expression level.

### 4.5. The miRNA Profiling

A microarray system GeneChip miRNA 4.0 Array chip, (Affymetrix, Santa Carla, CA, USA) was used to determine the miRNA expression profiles. The RNA preparation and hybridization were performed according to the manufacturer’s protocol. High-quality RNA (RNA integrity number >7.5) was used for expression microarray analysis. The arrays were scanned using the Affymetrix Gene Chip Scanner 3000 7G System with Workstation and AutoLoader (Affymetrix, Santa Carla, CA, USA), and CEL. intensity files were generated by Affymetrix GeneChip Command Console Software 3.1.4 (AGCC, Affymetrix, Santa Carla, CA, USA).

The Affymetrix GeneChip miRNA 4.0 microarrays were analyzed as log2-transformed intensities using the Affymetrix Transcriptome Analysis Console 4.0.2 (TAC, Affymetrix, Santa Carla, CA, USA), following the software’s guidelines to determine differentially expressed genes (DEGs) between the CRC and control probes. The significant genes (*p*-value < 0.05) were ranked by fold-change with a cutoff of 4. Expression analysis of the studied genes was performed using ExpressionSuite Software Version 1.3 and StepOne Software v2.2.2.

### 4.6. Statistical Analysis

Statistical analysis of the obtained data was performed using Statistica v.13.3 software with the following tests: to evaluate the normality of distribution of Shapiro–Wilk, and to assess differences between study groups: U-Mann–Whitney and Kruskal–Wallis ANOVA. The level of statistical significance was taken at *p* < 0.05.

## 5. Conclusions

As a result of the study, we observed that the tissue surrounding the colorectal tumor in patients with advanced cancer and metastases to distant organs showed a significantly higher expression of miR-3613-3p, which suggests a protective function of the surrounding tissue, taking into account the effect of miR-3613-3p suggested in the literature as a tumor suppressor. In addition, in our studies, we show that radiotherapy probably contributes to the increase in the expression of miR-3613-3p both in tumor cells and in the surrounding tissue, which indicates the possibility of considering this microRNA as a potential therapeutic target. Due to the ambiguous role of miR-3613-3p in the pathomechanism of colorectal cancer, further studies are needed, taking into account the survival time of patients and response to treatment in correlation with miR-3613-3p expression.

## Figures and Tables

**Table 1:** Difference in mean expression of miR-3613-3p depending on the analyzed group: control, tumor, surrounding tissue, benign tumor, tissue surrounding benign tumor, * *p* < 0.05 Kruskal–Wallis ANOVA.

Group	N	Mean	SE	*p*-Value				
				Control	Tumor	Surrounding Tissue	Benign Tumor	Tissue Surrounding a Benign Tumor
Control	13	10.420	4.870		0.0020 *	0.302	0.622	0.131
Tumor	60	0.559	0.144	0.002 *		0.00003 *	0.020 *	0.0004 *
Surrounding tissue	60	17.278	3.273	0.302	0.00003 *		0.101	0.037 *
Benign tumor	5	6.674	6.592	0.622	0.0200 *	0.101		0.026 *
Tissue surrounding a benign tumor	5	73.591	45.746	0.131	0.0004 *	0.037 *	0.026 *	

**Table 2:** Characteristics of the study group in terms of TNM clinical classification.

Variable	N	%
cT stage		
T2	7	11
T2/T3	3	5
T3	34	52
T4	16	24
T4a	1	2
unknown	4	6
cN stage		
N0	8	13
N1	7	11
N2	5	7
Nx	43	66
Nx/0	1	2
unknown	7	11
cM stage		
M0	52	80
M1	6	9
Mx/0	1	2
unknown	6	9

**Table 3:** Characteristics of the study group in terms of TNM pathological classification.

Variable	N	%
pT stage		
T1	2	2.5
T2	14	21
T3	42	65
T4	6	9
T4a	2	2.5
pN stage		
N0	31	48
N1	10	15
N1a	3	5
N1b	7	11
N2	3	5
N2a	5	7
N2b	6	9
pM stage		
M0	56	86
M1	5	7
M1a	3	5
M1b	1	2

**Table 4:** Characteristics of the study group in terms of tumor location.

Variable	N	%
Organ		
rectum	27	42
hepatic fold	5	8
caecum	5	8
splenic fold	3	4
sigmoid	12	19
descending colon	5	8
transverse colon	7	11

**Table 5:** Characteristics of the study group in terms of tumor advancement based on TNM.

Variable	N	%
Advancement according to TNM		
I	14	21
IIA	14	21
IIB	2	3
IIIA	2	3
IIIB	23	36
IIIC	2	3
IV	5	7
IVA	2	3
IVB	2	3

## Data Availability

The data used to support the findings of this study are included in the article.

## Supplementary Materials

The following supporting information can be downloaded at: https://www.mdpi.com/article/10.3390/ijms241814023/s1.
