# Supplementary material for: Influence of Clinical Factors on miR-3613-3p Expression in Colorectal Cancer
Source: Int J Mol Sci. 2023 Sep 13;24(18):14023. doi: 10.3390/ijms241814023 (PMC10531160; doi:10.3390/ijms241814023)
Supplement: Supplementary file 1 [file ijms-24-14023-s001.zip › ijms-2562708-supplementary.pdf]

Parameter	Surrounding tissue							Tumor						
	N	Mean	SD	N	Mean	SD	p-value	N	Mean	SD	N	Mean	SD	p-value
expression miR-3613-3p (RQ)	**cT stage**							**cT stage**						
	**T1+T2**			**T3+T4**				**T1+T2**			**T3+T4**			
	10	16.668	27.213	51	18.713	20.630	0.833	10	0.294	0.2628	51	0.632	1.0235	0.396
	**cN stage**							**cN stage**						
	**N0**			**N1+N2**				**N0**			**N1+N2**			
	8	18.576	26.353	12	23.906	13.226	0.667	8	0.304	0.213	12	0.370	0.254	0.623
	**pT stage**							**pT stage**						
	**T1+T2**			**T3+T4**				**T1+T2**			**T3+T4**			
	16	19.927	23.211	50	16.423	20.520	0.652	16	0.314	0.259	50	0.638	1.034	0.361
	**pN stage**							**pN stage**						
	**N0**			**N1 + N2**				**N0**			**N1 + N2**			
	31	13.692	18.045	34	20.693	23.329	0.291	31	0.635	1.070	34	0.502	0.794	0.657
	**cM stage**							**cM stage**						
	**M0**			**M1**				**M0**			**M1**			
	52	16.020	17.871	6	46.265	41.951	0.016	52	0.632	1.008	6	0.228	0.236	0.436
	**pM stage**							**pM stage**						
	**M0**			**M1**				**M0**			**M1**			
	56	15.683	17.729	9	28.761	38.149	0.195	56	0.625	0.971	9	0.183	0.195	0.278
	**Advancement according to the TNM scale**							**Advancement according to the TNM scale**						
	**I + II**			**III + IV**				**I + II**			**III + IV**			
	30	13.692	18.045	35	20.693	23.329	0.291	30	0.622	1.042	35	0.502	0.794	0.681
